# Trans-activation of eotaxin-1 by Brg1 contributes to liver regeneration

**DOI:** 10.1038/s41419-022-04944-0

**Published:** 2022-05-25

**Authors:** Zhiwen Fan, Ming Kong, Wenhui Dong, Chunlong Dong, Xiulian Miao, Yan Guo, Xingyu Liu, Shuying Miao, Lin Li, Tingting Chen, Yeqing Qu, Fei Yu, Yunfei Duan, Yunjie Lu, Xiaoping Zou

**Affiliations:** 1 Department of Pathology, Nanjing Drum Tower Hospital Affiliated with Nanjing University School of Medicine, Nanjing, China; 2 Department of Gastroenterology, Nanjing Drum Tower Hospital Affiliated with Nanjing University School of Medicine, Nanjing, China; 3 Key Laboratory of Targeted Intervention of Cardiovascular Disease and Collaborative Innovation Center for Cardiovascular Translational Medicine, Department of Pathophysiology, Nanjing Medical University, Nanjing, China; 4 Department of Hepatobiliary Surgery, Nanjing Drum Tower Hospital, Clinical College of Traditional Chinese and Western Medicine, Nanjing University of Chinese Medicine, Nanjing, China; 5 College of Life Sciences and Institute of Biomedical Research, Liaocheng University, Liaocheng, China; 6 Experimental Animal Center, Nanjing Drum Tower Hospital Affiliated with Nanjing University School of Medicine, Nanjing, China; 7 Department of Hepatobiliary Surgery, the First People’s Hospital of Changzhou, the Third Hospital Affiliated with Soochow University, Changzhou, China

**Keywords:** Transcriptional regulation, Liver regeneration, Eosinophil chemotaxis, Chemokine, Chromatin remodeling protein, Transcriptional regulatory elements, Epigenetics, Liver diseases, Chemotaxis

## Abstract

Infiltration of eosinophils is associated with and contributes to liver regeneration. Chemotaxis of eosinophils is orchestrated by the eotaxin family of chemoattractants. We report here that expression of eotaxin-1 (referred to as eotaxin hereafter), but not that of either eotaxin-2 or eotaxin-3, were elevated, as measured by quantitative PCR and ELISA, in the proliferating murine livers compared to the quiescent livers. Similarly, exposure of primary murine hepatocytes to hepatocyte growth factor (HGF) stimulated eotaxin expression. Liver specific deletion of Brahma-related gene 1 (Brg1), a chromatin remodeling protein, attenuated eosinophil infiltration and down-regulated eotaxin expression in mice. Brg1 deficiency also blocked HGF-induced eotaxin expression in cultured hepatocytes. Further analysis revealed that Brg1 could directly bind to the proximal eotaxin promoter to activate its transcription. Mechanistically, Brg1 interacted with nuclear factor kappa B (NF-κB)/RelA to activate eotaxin transcription. NF-κB knockdown or pharmaceutical inhibition disrupted Brg1 recruitment to the eotaxin promoter and blocked eotaxin induction in hepatocytes. Adenoviral mediated over-expression of eotaxin overcame Brg1 deficiency caused delay in liver regeneration in mice. On the contrary, eotaxin depletion with RNAi or neutralizing antibodies retarded liver regeneration in mice. More important, Brg1 expression was detected to be correlated with eotaxin expression and eosinophil infiltration in human liver specimens. In conclusion, our data unveil a novel role of Brg1 as a regulator of eosinophil trafficking by activating eotaxin transcription.

## Introduction

Liver regeneration is a pathophysiologically significant process that facilitates recovery of liver function from various injuries. Insufficient liver regeneration is a major underlying cause of liver failure [1]. Liver regeneration is primarily mediated by hepatocytes, which exit from the quiescent state and resume active proliferation in response to a wide range of stimuli [2]. However, the ability of the injured liver to rejuvenate itself is hardly is a hepatocyte-autonomous process. Instead, multiple cell lineages, including hepatic stellate cells, sinusoidal endothelial cells (LSECs), and immune cells, both intrahepatic and circulating, contribute to liver regeneration [3]. Pioneering work by Jacquelyn Maher demonstrated that LSECs, as opposed to other hepatic cell types, represent the major source of hepatocyte growth factor, essential for hepatocyte to re-enter the cell cycle, during liver regeneration [4]. Because LSECs are believed to originate from bone marrow sinusoidal progenitor cells (BM SPCs), depletion of these cells through irradiation severely retards liver regeneration in vivo [5]. Macrophages can also regulate hepatocyte proliferation and liver regeneration although the precise role remains controversial. Boulton et al have shown that depletion of resident hepatic macrophages with dichlormethylene bisphosphonate augmented liver regeneration following partial hepatectomy (PHx) in rats [6]. On the contrary, Abshagen et al, using the same strategy, reported that macrophage depletion delayed liver regeneration in mice with liver resection [7]. The contribution of natural killer cells (NKCs) to liver regeneration is equally equivocal because there is evidence to suggest both a pro-regenerative role [8] and an anti-regenerative role [9] for this pool of cells.

Eosinophils are characterized as a sub-population of bone marrow-derived granulocytes [10]. Initially considered to be a prominent mediator of specialized host defense response such as helminth infection [11, 12] and allergy [13], mounting evidence points to far more encompassing roles for eosinophils in the maintenance of internal homeostasis including the liver [14, 15]. Under physiological conditions, eosinophils are under-represented in the liver constituting ~1% total myeloid cells [16]. In response to various injurious cues, eosinophils migrate to and accumulate in the liver to promote liver regeneration [17]. Depletion of eosinophils with a specific neutralizing antibody impairs liver regeneration likely owing to disruption of IL-4 secretion. In addition, Ju and colleagues have observed an accumulation of eosinophils in human liver grafts after hepatic transplantation, which could potentially contribute to alleviation of ischemia-reperfusion injury by boosting liver regeneration through an ST2-IL-13 pathway [15]. On the other hand, eosinophils appear to play a key role in halothane-induced liver injury: eosinophil-depleting antibodies or deficiency in the lineage specific transcription factor GATA1 (ΔdblGata^−/−^) attenuated liver hepatic necrosis. However, it is not clear whether liver regeneration was altered in this model [14]. It should also be noted that eosinophils have been shown to promote a regenerative response in the heart [18], in the pulmonary epithelium [19], in the corneal epithelium [20], and in the muscle [21]. Eosinophil trafficking is steered by the eotaxin family of chemokines consisting of eotaxin-1 (CCL11, hereafter referred to as eotaxin), eotaxin-2 (CCL24), and eotaxin-3 (CCL26). First isolated and characterized by the Williams laboratory, eotaxin is a polypeptide of 73 amino acids [22]. Several studies have since demonstrated the essentiality of eotaxin in eosinophil attraction and disease pathogenesis. For instance, Fulkerson et al have reported that deletion of eotaxin in mice blocked allergen induced pulmonary eosinophilia and airway inflammation [23]. Rosenberg et al have shown that disruption of eotaxin expression in mice influences the early phase, but not the late phase, of eosinophil migration to the airway and to the skin in two separate animal models [24]. Elevation of plasma eotaxin levels has been observed in patients with chronic liver disease [25] and drug-induced liver disease [26]. Regulation of eotaxin expression in the context of liver regeneration has yet to be explored.

Brg1 is a component of the mammalian SWI/SNF chromatin remodeling complex and abundantly expressed in different cell types in the liver. The involvement of Brg1 in various liver injuries has been extensively studied in our laboratory. Deletion of Brg1 in hepatocytes has been shown to mitigate the pathogenesis of non-alcoholic steatohepatitis [27–31], and fulminant hepatitis [32, 33]. Endothelial-specific Brg1 deletion, on the other hand, is associated with attenuation of liver fibrosis [34–36]. Previously we have shown that Brg1 deficiency leads to impaired liver regeneration in mice attributable to the disruption of Wnt-β-catenin signaling and delayed hepatocyte proliferation [37]. In the present study, we investigated the transcriptional regulation of eotaxin by Brg1. Our data suggest that Brg1 interacts with NF-κB/p65 to activate eotaxin transcription in hepatocytes thus promoting eosinophil homing.

## Methods

### Animals

All animal protocols were reviewed and approved the intramural Ethics Committee on Humane Treatment of Laboratory Animals of Nanjing Medical University (approval reference #: IACUC-1811060). The mice were maintained in an SPF environment with 12 h light/dark cycles and libitum access to food and water. Hepatocyte conditional Brg1 knockout (Brg1^LKO^) mice have been described previously [30]. Liver regeneration by the partial hepatectomy (PHx) procedure or intraperitoneal injection of acetaminophen (APAP) as previously described [37]. Typically, 6-wk male mice (20–22 g) were used for the PHx experiments whereas 8-wk male mice (25–27 g) were used for the APAP experiments.

### Cells, transient transfection, and reporter assay

Human hepatoma cells (HepG2) were maintained in DMEM supplemented with 10% FBS. Human eosinophilic leukaemia cells (EoL-1) were maintained in RPMI 1640 supplemented with 2 mM glutamine and 10% FBS. Primary murine hepatocytes were isolated as previously described [38] and seeded at 2 × 10^5^ cells/well for 12-well culture dishes, or 4 × 10^5^ cells/well for 6-well culture dishes, or 4 × 10^6^ cells/well for p100 culture dishes. Cell viability was examined at the time of seeding by trypan blue staining; typical isolation yielded >95% viability. Mouse recombinant HGF and mouse recombinant eotaxin were purchased from R&D. EVP4593 was purchased from Selleck. BRG1 expression construct [39, 40] and *Eotaxin* promoter-luciferase constructs [41] have been previously described. Small interfering RNAs were purchased from GenePharma. Transient transfections were performed with Lipofectamine 2000. Luciferase activities were assayed 24–48 h after transfection using a luciferase reporter assay system (Promega) as previously described [42, 43].

### Protein extraction and Western blot

Whole cell lysates were obtained by re-suspending cell pellets in RIPA buffer (50 mM Tris pH7.4, 150 mM NaCl, 1% Triton X-100) with freshly added protease inhibitor (Roche) as previously described [44, 45]. Typically, 100μl RIPA buffer was used for 1 × 10^6^ cells. 30 μg of protein were loaded in each lane and separated by 8% PAGE-SDS gel with all-blue protein markers (Bio-Rad). Proteins were transferred to nitrocellulose membranes (Bio-Rad) in a Mini-Trans-Blot Cell (Bio-Rad). The membranes were blocked with 5% fat-free milk powder in Tris-buffered saline (TBS) at room temperature for half an hour and then incubated with the following primary antibodies at 4°C overnight: anti-NF-κB/p65 (Cell Signaling Tech, 8242, 1:1000), anti-phopho-NF-κB/p65 (Cell Signaling Tech, 3033, 1:1000), and anti-β-actin (Sigma, A1978, 1:5000). The next day, the membranes were washed with TBS and incubated with HRP conjugated anti-rabbit secondary antibody (Thermo Fisher, 61-6520, 1:5000) or anti-mouse secondary antibody (Thermo Fisher, 31464, 1:5000) for one hour at room temperature. For densitometrical quantification, densities of target proteins were normalized to those of β-actin. Data are expressed as relative protein levels compared to the control group which is arbitrarily set as 1. Full-scan, uncropped blots are presented as Fig.S1 in the online supplementary material.

### RNA isolation and Real-time PCR

RNA was extracted with the RNeasy RNA isolation kit (Qiagen); typically, 300μl lysis buffer was used for 0.5 × 10^6^ cells. Reverse transcriptase reactions were performed using a SuperScript First-strand Synthesis System (Invitrogen) as previously described [35, 46]; typically, 1μg RNA was used for each reverse transcriptase reaction in a 20 μl system. Real-time PCR reactions were performed on an ABI Prism 7500 system with the following primers: *Eotaxin*, 5ʹ-GAATCACCAACAACAGATGCAC-3ʹ and 5ʹ-ATCCTGGACCCACTTCTTCTT-3ʹ; *Eotaxin-2*, 5ʹ-ATTCTGTGACCATCCCCTCAT-3ʹ and 5ʹ-TGTATGTGCCTCTGAACCCAC-3ʹ; *Eotaxin-3*, 5ʹ-TTCTTCGATTTGGGTCTCCTTG-3ʹ and 5ʹ-GTGCAGCTCTTGTCGGTGAA-3ʹ. Ct values of target genes were normalized to the Ct values of housekeekping control gene (18 s, 5ʹ-CGCGGTTCTATTTTGTTGGT-3ʹ and 5ʹ-TCGTCTTCGAAACTCCGACT-3ʹ for both human and mouse genes) using the ΔΔCt method and expressed as relative mRNA expression levels compared to the control group which is arbitrarily set as 1.

### Enzyme-linked immunosorbent assay

Secreted Eotaxin levels were examined by ELISA as previously described using commercially available kits (for eotaxin/CCL11, R&D, catalog# MME00; for IL-4, R&D, catalog# M4000B; for IL-10, R&D, catalog#M1000B; for IL-12, R&D, catalog#M1270) according to vendor’s recommendations. For measuring eotaxin levels in the liver, cut a small slice of the tissue (~100 μg) and homogenize in ice-cold PBS with freshly added protease inhibitor cocktail (Sigma, P-8340). Centrifuge at 14000 g at 4 °C for 15 min. Remove and aliquot the supernatant for ELISA. Data were normalized by cell number (for supernatant collected from primary cell culture) or tissue weight (for liver homogenates).

### Chromatin immunoprecipitation (ChIP)

Chromatin Immunoprecipitation (ChIP) assays were performed essentially as described before [47, 48]. In brief, chromatin in control and treated cells were cross-linked with 1% formaldehyde. Cells were incubated in lysis buffer (150 mM NaCl, 25 mM Tris pH 7.5, 1% Triton X-100, 0.1% SDS, 0.5% deoxycholate) supplemented with protease inhibitor tablet and PMSF. DNA was fragmented into ~200 bp pieces using a Branson 250 sonicator. Aliquots of lysates containing 200 μg of protein were used for each immunoprecipitation reaction with 5μg of anti-BRG1 (Abcam, Ab110641), anti-NF-κB/p65 (Cell Signaling Tech, 8242), or pre-immune IgG. Precipitated DNA was amplified with the following primers: primer #1, 5ʹ-AAATGTACCAAGTCCCTCC-3ʹ and 5ʹ-ATGAGCAGCAGCCACAGAAG-3ʹ; primer #2, 5ʹ-ATCCTGAATTCAGGTTCTAC-3ʹ and 5ʹ-ATTCTGAGGAGTTGAC-3ʹ.

### Histology

Histological analyses were performed essentially as described before [49, 50]. Paraffin sections were stained with were blocked with 10% normal goat serum for 1 h at room temperature and then incubated with anti-eosinophil cationic protein (ECP, Proteinch, 55338-1, 1:200), anti-BRG1 (Proteintech, 21634-1, 1:200), or anti-Eotaxin (Genetex, 64437, 1:200) antibodies. Staining was visualized by incubation with HRP-conjugated anti-rabbit secondary antibody (Thermo Fisher, 31458, 1:2000) and developed with a streptavidin-horseradish peroxidase kit (Pierce) for 20 min. Pictures were taken using an Olympus IX-70 microscope. Quantifications were performed with Image J. For each mouse, at least three slides were stained and at least five different fields were analyzed for each slide.

### Eosinophil migration assay

Eosinophil migration was measured using the Boyden chamber inserts (5 μm, Corning). Briefly, human eosinophilic leukemia cells EoL-1 [51] were added to the upper chamber whereas the conditioned media collected from hepatocytes were added to the lower chamber. In certain experiments, recombinant murine eotaxin (20 ng/ml, R&D) was directly added to the conditioned media. The number of migrated EoL-1 cells in the lower chamber was counted in five randomly chosen fields using an inverted microscope.

### Human ALF specimens

Liver biopsies were collected from patients with ALF referring to Nanjing Drum Tower Hospital. Written informed consent was obtained from subjects or families of liver donors. All procedures that involved human samples were approved by the Ethics Committee of the Nanjing Drum Tower Hospital (approval reference #: 2020-155-01) and adhered to the principles outlined in the Declaration of Helsinki. Basic information for the patients is listed in supplementary Table I. Paraffin sections were stained with indicated antibodies. Slides were observed under a light microscope at high power (X40) by two pathologists independently in a double-blind fashion. The scoring system was based on the following criterion: the staining intensity was divided into five quantiles; the slides with the strongest staining were given a score of 5 (top quintile) whereas the slides with the dimmest staining were given a score of 1 (bottom quintile). For correlation analysis, the averages of BRG1 staining-score, EOTAXIN staining-score, and ECP staining-score for each sample were assessed for correlation coefficient.

### Statistical analysis

One-way ANOVA with post-hoc Scheff´e analyses were performed by SPSS software (IBM SPSS v18.0, Chicago, IL, USA). Unless otherwise specified, values of *p* < 0.05 were considered statistically significant.

## Results

### Eotaxin upregulation accompanies liver regeneration in mice

**Fig. 1:**
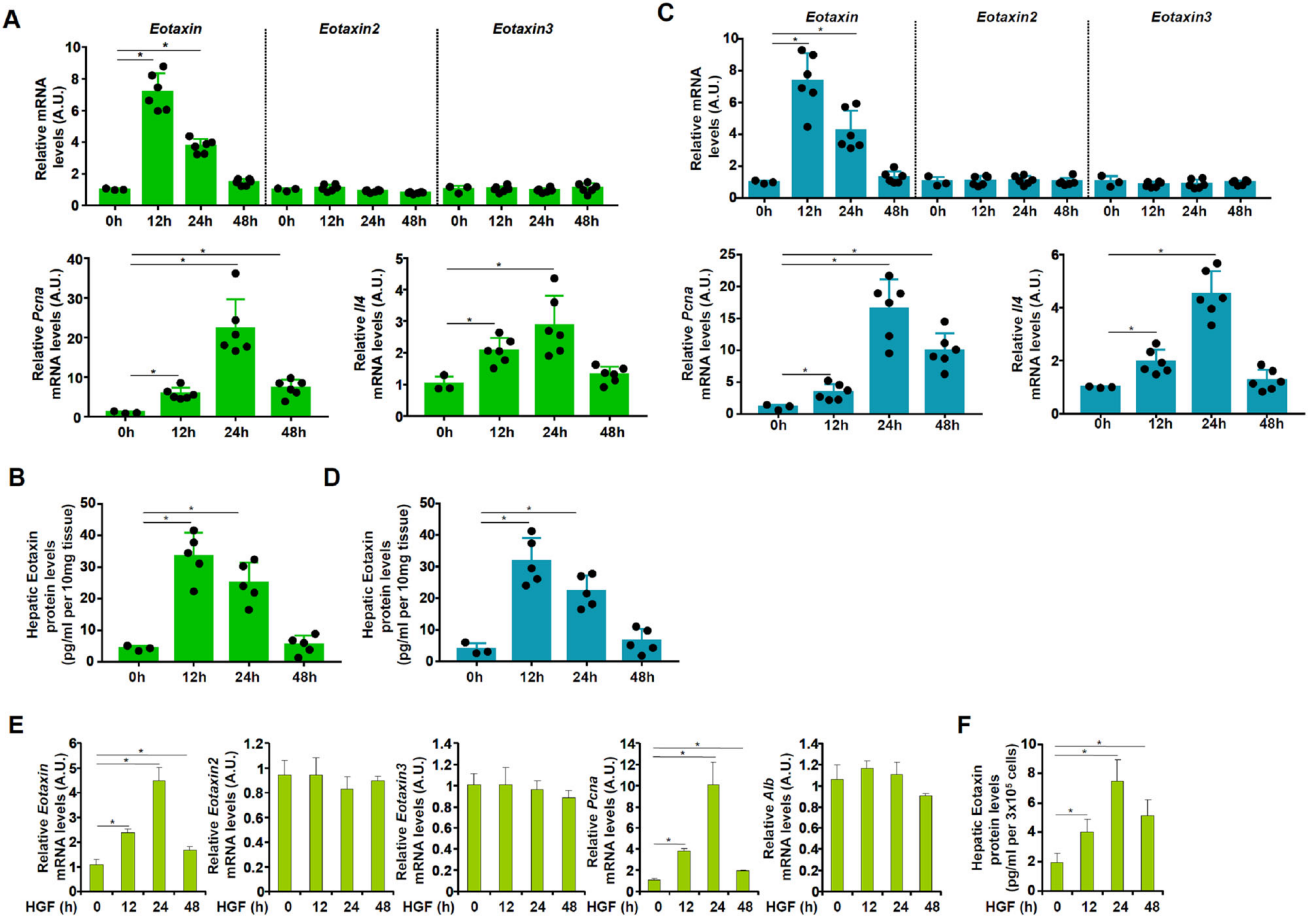
Eotaxin up-regulation accompanies liver regeneration in mice. **A**, **B**) C57B6/L mice were subjected to 2/3 PHx and sacrificed at indicated time points. Eotaxin expression levels were evaluated by qPCR and ELISA. *N* = 3–6 mice for each group. **C**, **D** C57B6/L mice were injected with APAP (300 mg/kg) and sacrificed at indicated time points. Eotaxin expression levels were evaluated by qPCR and ELISA. *N* = 3–6 mice for each group. **E**, **F** Primary murine hepatocytes were treated with HGF (20 ng/ml) and harvested at indicated time points. Eotaxin expression levels were evaluated by qPCR and ELISA. Error bars represent SD (*p < 0.05, one-way ANOVA). All experiments were repeated three times and one representative experiment is shown.

Because hepatic eosinophil abundance was reported to be appreciably increased in the process of liver regeneration [17], we hypothesized that there might be a concomitant increase in eotaxin levels. To test this hypothesis, partial hepatectomy (2/3 PHx) was performed in C57B6/L mice. When the mice were sacrificed at different time points after the surgery and expression levels of three different eotaxin isoforms were examined by qPCR, it was discovered that eotaxin, but not eotaxin-2 or eotaxin-3, was significantly up-regulated in the early phase of liver regeneration (12 h), declined at 24 h, and then returned to basal level at 48 h (Fig. 1A). Of note, the changes in eotaxin-1 expression correlated well with the proliferation marker PCNA and the eosinophil-derived cytokine IL-4 (Fig. 1A). ELISA data confirmed that there was a quick and transient elevation of eotaxin protein levels in the liver following 2/3 PHx (Fig. 1B). We then tried a second model of liver regeneration in which C57B6/L mice were injected with a single dose of acetaminophen (APAP) to induce acute liver injury. Similarly, we detected a rapid elevation in eotaxin, but neither eotaxin-2 nor eotaxin-3, mRNA expression in liver following APAP injection (Fig. 1C). Likewise, eotaxin protein levels, as measured by ELISA, mirrored the changes in eotaxin mRNA levels (Fig. 1D). Next, we treated primary murine hepatocytes with HGF, a prototypical pro-proliferative growth factor, and found that eotaxin levels were rapidly increased along with PCNA (Figs. 1E, F).

### Brg1 deficiency attenuates eosinophil infiltration in vivo and in vitro

**Fig. 2:**
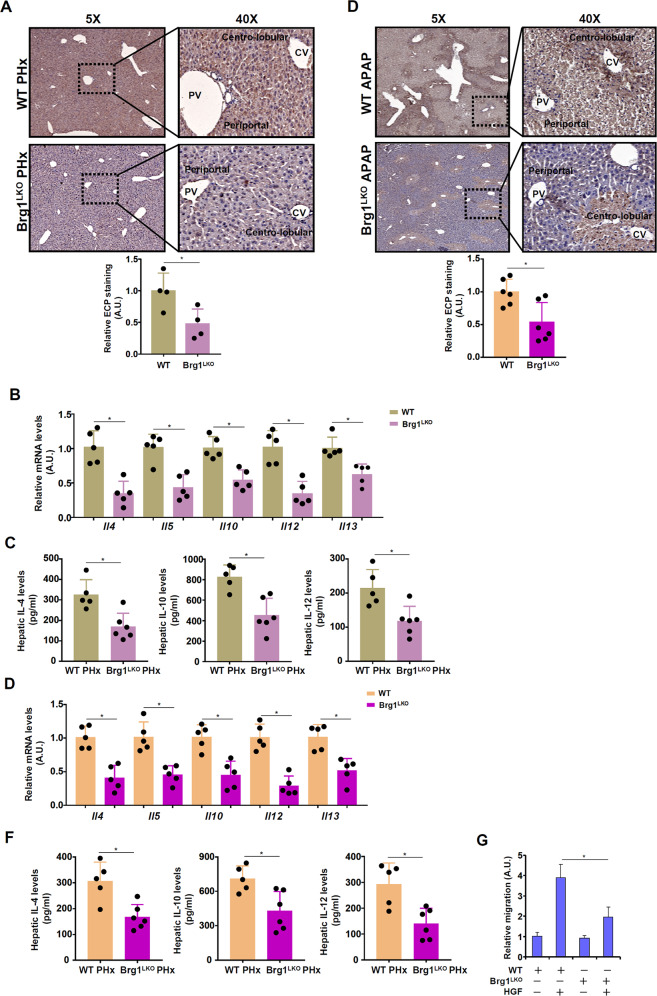
Brg1 deficiency attenuates eosinophil infiltration in vivo and in vitro. **A** WT and Brg1^LKO^ mice were subjected to 2/3 PHx and sacrificed 24 h after the surgery. Immunohistochemical staining was performed with anti-RNASE3/ECP. *N* = 4 mice for each group. **B**, **C** WT and Brg1^LKO^ mice were subjected to 2/3 PHx and sacrificed 24 h after the surgery. Eosinophil-derived cytokines were examined by qPCR and ELISA. *N* = 5 mice for each group. **D** WT and Brg1^LKO^ mice were injected with APAP (300 mg/kg) and sacrificed 24 h after the injection. Immunohistochemical staining was performed with anti-RNASE3/ECP. *N* = 6 mice for each group. **E**, **F** WT and Brg1^LKO^ mice were injected with APAP (300 mg/kg) and sacrificed 24 h after the injection. Eosinophil-derived cytokines were examined by qPCR and ELISA. *N* = 5 mice for each group. **G** Primary hepatocytes isolated from WT and Brg1^LKO^ mice were treated with or without HGF (20 ng/ml) for 24 h. Conditioned media were collected and eosinophil migration assay was performed as described in Methods. Error bars represent SD (*p < 0.05, one-way ANOVA). All experiments were repeated three times and one representative experiment is shown.

We [37] and others [52] have previously reported that the chromatin remodeling protein Brahma related gene 1 (Brg1) plays an essential role in liver regeneration. Immunohistochemical staining of hepatic sections from wild type (WT) and Brg1 liver conditional knockout (Brg1^LKO^) mice subjected to partial hepatectomy with an anti-eosinophil cationic protein (ECP) antibody, which recognizes an eosinophil specific surface marker, revealed that eosinophil infiltration was significantly dampened in the Brg1^LKO^ livers compared to the WT livers (Fig. 2A). Consistent with this observation, hepatic levels of several eosinophil-specific cytokines, including interleukin 4 (Il-4), Il-5, Il-10, Il-12, and Il-13, were collectively down-regulated in the Brg1^LKO^ mice compared to the WT mice following PHx (Figs. 2B, C). Similar observations were made in the APAP model (Fig. 2D–F), which led us to conclude that Brg1 deficiency in hepatocytes might interfere with eosinophil infiltration during liver regeneration. To further authenticate this proposal, primary hepatocytes were isolated from the WT mice and the Brg1^LKO^ mice and treated with HGF. Conditioned media collected from the WT hepatocytes exposed to HGF treatment elicited stronger eosinophil migration than those from the Brg1^LKO^ hepatocytes (Fig. 2G).

### Brg1 deficiency down-regulates eotaxin expression in vivo and in vitro

**Fig. 3:**
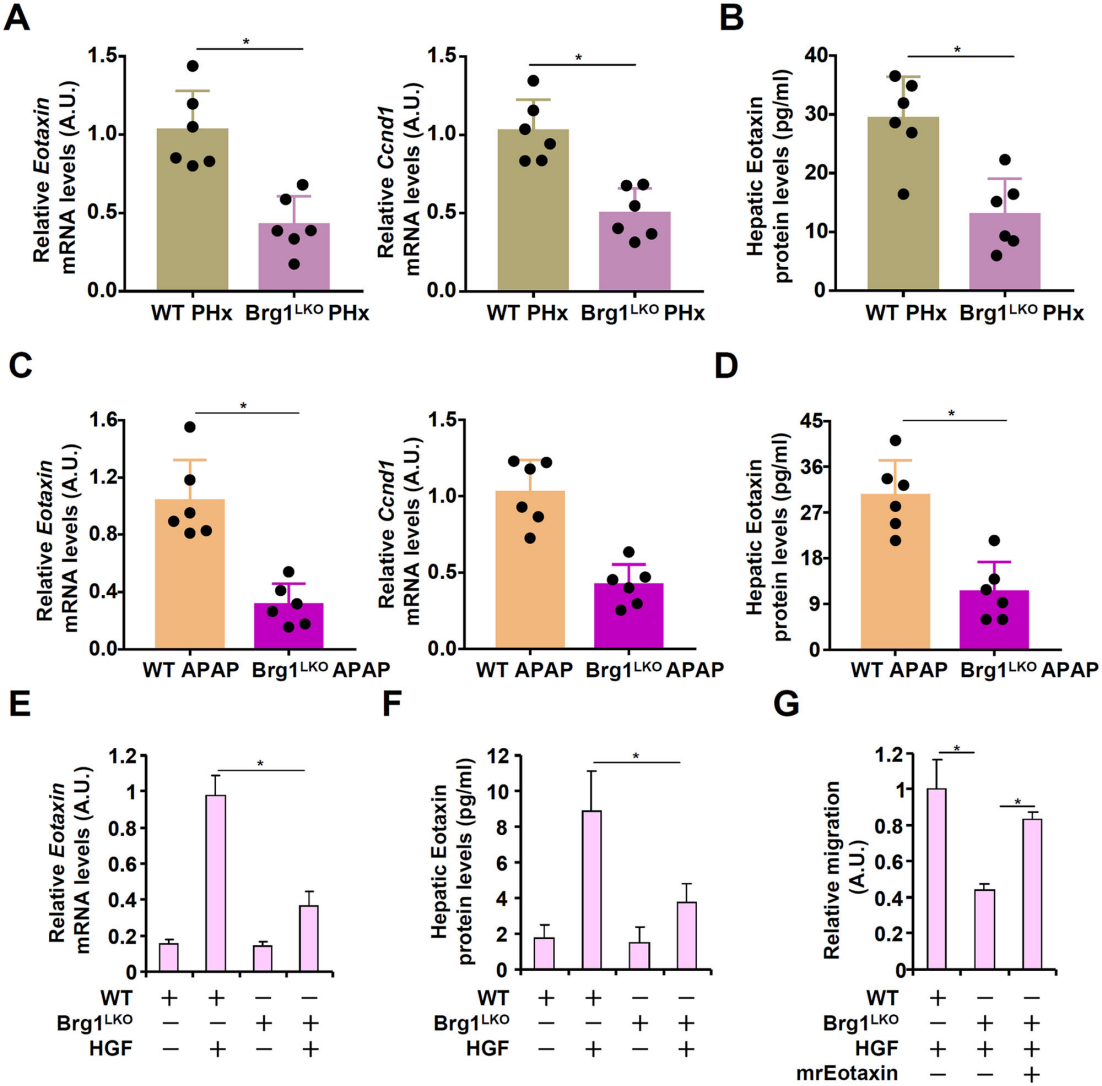
Brg1 deficiency down-regulates eotaxin expression in vivo and in vitro. **A**, **B** WT and Brg1^LKO^ mice were subjected to 2/3 PHx and sacrificed 24 h after the surgery. Eotaxin expression levels were evaluated by qPCR and ELISA. *N* = 6 mice for each group. **C**, **D** WT and Brg1^LKO^ mice were injected with APAP (300 mg/kg) and sacrificed 24 h after the injection. Eotaxin expression levels were evaluated by qPCR and ELISA. Each dot in the scatter plot represents a single mouse. *N* = 6 mice for each group. **E**, **F** Primary hepatocytes isolated from WT and Brg1^LKO^ mice were treated with or without HGF (20 ng/ml) for 24 h. Eotaxin expression levels were evaluated by qPCR and ELISA. **G** Primary hepatocytes isolated from WT and Brg1^LKO^ mice were treated with or without HGF (20 ng/ml) for 24 h. Conditioned media were collected and eosinophil migration assay was performed in the presence or absence of recombinant eotaxin. Error bars represent SD (*p < 0.05, one-way ANOVA). All experiments were repeated three times and one representative experiment is shown.

Because Brg1 deficiency was associated with amelioration of eosinophil infiltration in the liver during liver regeneration, we examined the possibility that Brg1 might contribute to the induction of eotaxin expression. As shown in Fig. 3A and Fig. 3B, eotaxin mRNA expression and eotaxin protein expression was down-regulated by 58 and 61%, respectively, in the Brg1^LKO^ livers compared to the WT livers following 2/3 PHx. Similarly, there was a 70% decrease in eotaxin mRNA expression (Fig. 3C) and a 62% decrease in eotaxin protein levels (Fig. 3D) in the Brg1^LKO^ livers compared to the WT livers following APAP injection. Similarly, when primary hepatocytes were isolated from the Brg1^LKO^ livers and treated with HGF, induction of eotaxin expression was not as strong as in the cells isolated from the WT livers (Figs. 3E, F). More important, the addition of recombinant eotaxin (mrEotaxin) in the conditioned media collected from the Brg1^LKO^ hepatocytes corrected the deficiency of promoting eosinophil migration (Fig. 3G).

### Brg1 directly binds to the eotaxin promoter and activates eotaxin transcription

**Fig. 4:**
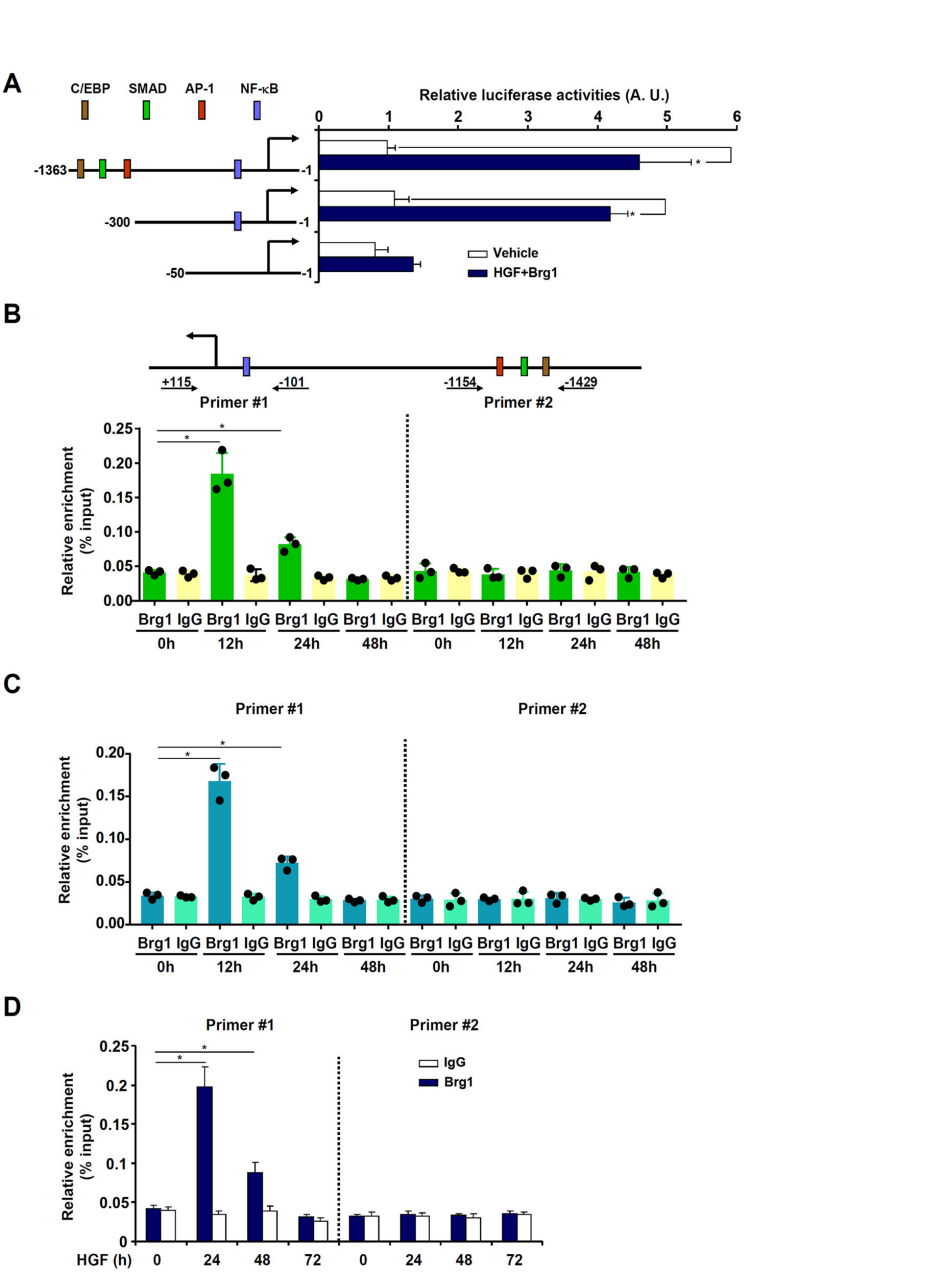
Brg1 directly binds to the eotaxin promoter and activates eotaxin transcription. **A** Eotaxin promoter constructs harboring serial deletions were co-transfected with a Brg1 expression vector into HepG2 cells followed by treatment with HGF (20 ng/ml) for 24 h. Luciferase activities were normalized by protein concentration and GFP fluorescence. **B** C57B6/L mice were subjected to 2/3 PHx and sacrificed at indicated time points. ChIP assay was performed with anti-Brg1 or IgG. Each dot in the scatter plot represents a single mouse. **C** C57B6/L mice were injected with APAP (300 mg/kg) and sacrificed at indicated time points. ChIP assay was performed with anti-Brg1 or IgG. Each dot in the scatter plot represents a single mouse. **D** Primary murine hepatocytes were treated with HGF (20 ng/ml) and harvested at indicated time points. ChIP assay was performed with anti-Brg1 or IgG. Error bars represent SD (*p < 0.05, one-way ANOVA). All experiments were repeated three times and one representative experiment is shown.

We performed the following experiments to tackled the question as to whether Brg1-dependent induction of eotaxin expression occurred at the transcriptional level. First, a eotaxin promoter-luciferase reporter construct was co-transfected into HepG2 cells with or without a Brg1 expression vector followed by HGF treatment. Whereas Brg1 over-expression combined with HGF treatment augmented the activity of the two longer eotaxin promoter constructs (−1363 and −300), it failed to activate the shortest (−50) construct, suggesting that Brg1 might bind to the eotaxin promoter between −300 and −50 relative to the transcription start site (Fig. 4A). To verify Brg1 could indeed bind to the eotaxin promoter, chromatin immunoprecipitation (ChIP) assay was performed. The precipitated DNA was amplified by two different sets of primers: the first set of primers (#1, −101/+115) spanned the proximal eotaxin promoter region containing a conserved NF-κB site whereas the second set of primers (#2, −1429/−1154) spanned the distal eotaxin promoter containing motifs for the transcription factors C/EBP, SMAD, and AP-1. The results indicate that in the regenerative livers following 2/3 PHx (Fig. 4B) or APAP injection (Fig. 4C), Brg1 started to occupy the proximal eotaxin promoter (amplified by primer set #1), but not the distal eotaxin promoter (amplified by primer set #2), with a kinetics mirroring the induction of eotaxin expression. In primary murine hepatocytes, HGF treatment similarly stimulated the recruitment of Brg1 to the proximal eotaxin promoter, which peaked early (24 h) after the treatment and subsided at later time points (Fig. 4D).

### Brg1 interacts with NF-κB to activate eotaxin transcription

**Fig. 5:**
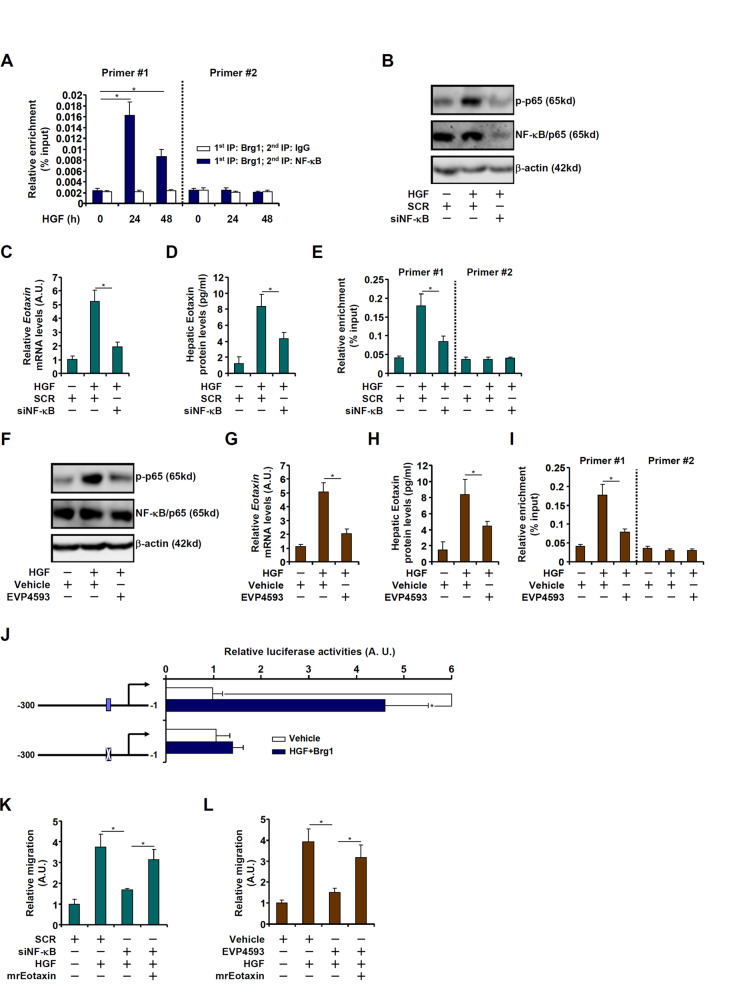
Brg1 interacts with NF-κB to activate eotaxin transcription. **A** Primary murine hepatocytes were treated with HGF (20 ng/ml) and harvested at indicated time points. Re-ChIP assay was performed with indicated antibodies. **B**–**E** Primary murine hepatocytes were transfected with siRNA targeting NF-κB or scrambled siRNA followed by treatment with HGF (20 ng/ml). NF-κB expression was examined by Western blotting. Eotaxin expression levels were evaluated by qPCR and ELISA. ChIP assay was performed with anti-Brg1. **F**–**I** Primary murine hepatocytes were treated with HGF (20 ng/ml) in the presence or absence of EVP4593 (100 nM). NF-κB expression was examined by Western blotting. Eotaxin expression levels were evaluated by qPCR and ELISA. ChIP assay was performed with anti-Brg1. **J** WT and NF-κB mutant Eotaxin promoter constructs were co-transfected with a Brg1 expression vector into HepG2 cells followed by treatment with HGF (20 ng/ml) for 24 h. Luciferase activities were normalized by protein concentration and GFP fluorescence. (**K**) Primary murine hepatocytes were transfected with siRNA targeting NF-κB or scrambled siRNA followed by treatment with HGF (20 ng/ml). Conditioned media were collected and eosinophil migration assay was performed as described in Methods. **L** Primary murine hepatocytes were treated with HGF (20 ng/ml) in the presence or absence of EVP4593 (100 nM). Conditioned media were collected and eosinophil migration assay was performed as described in Methods. Error bars represent SD (*p < 0.05, one-way ANOVA). All experiments were repeated three times and one representative experiment is shown.

There is a conserved NF-κB site between −300 and −50 on the eotaxin promoter [53]. Re-ChIP assay showed that a Brg1-NF-κB/p65 complex could be detected on the proximal eotaxin promoter in hepatocytes treated by HGF (Fig. 5A), pointing to the possibility that NF-κB might be essential for Brg1 recruitment and thus eotaxin induction. HGF treatment stimulated NF-κB activity as measured by NF-κB phosphorylation, which was blocked by siRNA-mediated NF-κB depletion (Fig. 5B). Consequently, induction of eotaxin expression, at both mRNA (Fig. 5C) and protein (Fig. 5D) levels, were dampened. This could be due to, at least in part, disturbed Brg1 binding to the eotaxin promoter (Fig. 5E). In an alternative set of experiments, NF-κB activity was inhibited by EVP4593 [54]. Treatment with EVP4593 decreased NF-κB phosphorylation (Fig. 5F), attenuated eotaxin induction (Figs. 5G, 5H), and weakened Brg1 binding to the eotaxin promoter (Fig. 5I). Finally, mutagenesis of the NF-κB site in the context of the −300 eotaxin promoter construct completely abrogated induction of the promoter by Brg1 over-expression plus HGF treatment (Fig. 5J). Consistent with its ability to regulate eotaxin expression, manipulation of NF-kB expression by siRNA (Fig. 5K) or activity by EVP4593 (Fig. 5L) resulted in diminished eosinophil migration, which could be partially rescued by the addition by recombinant eotaxin.

### Eotaxin restores liver regeneration in Brg1-null mice

**Fig. 6:**
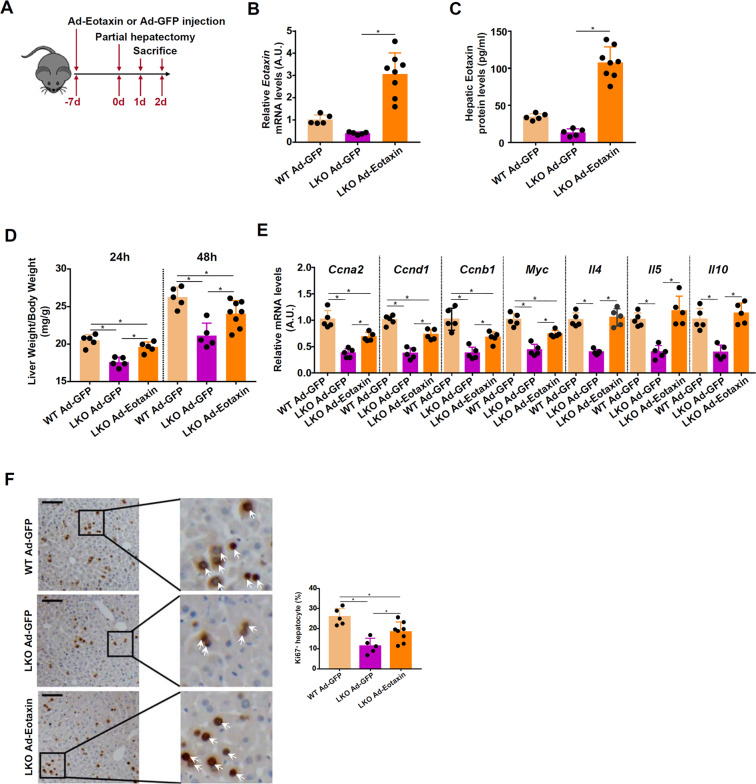
Eotaxin restores liver regeneration in Brg1-null mice. WT and Brg1^LKO^ mice were injected with adenovirus carrying eotaxin expression vector (Ad-Eotaxin) or control adenovirus (Ad-GFP) followed by 2/3 PHx. **A** Scheme of animal protocol. **B**, **C** Hepatic eotaxin expression was examined by qPCR and ELISA. **D** Liver weight versus body weight was calculated at 24 and 48 h post-surgery. **E** Expression levels of pro-proliferative genes in the liver samples collected at 48 h were examined by qPCR. **F** Paraffin embedded sections were stained with anti-Ki67. *N* = 5–8 mice for each group.

Next, the relevance of eotaxin in liver regeneration in vivo was investigated. WT and Brg1^LKO^ mice were injected via tail vein adenovirus carrying an eotaxin expression vector (Ad-Eotaxin) or a control vector (Ad-GFP) followed by PHx (Fig. 6A). Adenoviral mediated eotaxin over-expression more than compensated for the deficiency in eotaxin in the Brg1^LKO^ livers as measured by qPCR (Fig. 6B) and ELISA (Fig. 6C). Eotaxin replenishment partially restored liver regeneration as determined by liver weight/body weight at 24 and 48 h post-surgery (Fig. 6D). Expression levels of pro-proliferative genes, including *Ccna2*, *Ccnb1*, *Ccnd1*, and *Myc*, were conspicuously lower in the Ad-GFP injected Brg1^LKO^ mice but significantly higher in the Ad-Eotaxin injected Brg1^LKO^ mice (Fig. 6E). Of note, the levels of eosinophil-derived pro-regenerative cytokines (*Il4*, *Il5*, and *Il10*) in the Ad-Eotaxin-injected Brg1^LKO^ livers were indistinguishable from the WT livers although the changes were not as dramatic as eotaxin. It is possible that once eotaxin levels reach a certain threshold, its ability to recruit eosinophils may not be the rate-limiting step. It is equally possible that the ectopic eotaxin may not quite as biologically competent in terms of chemotaxis as the endogenous eotaxin. When proliferation of hepatocytes was measured by Ki67 staining, it was found that eotaxin over-expression similarly boosted liver regeneration in the Brg1^LKO^ mice (Fig. 6F).

### Eotaxin depletion impairs liver regeneration in mice

**Fig. 7:**
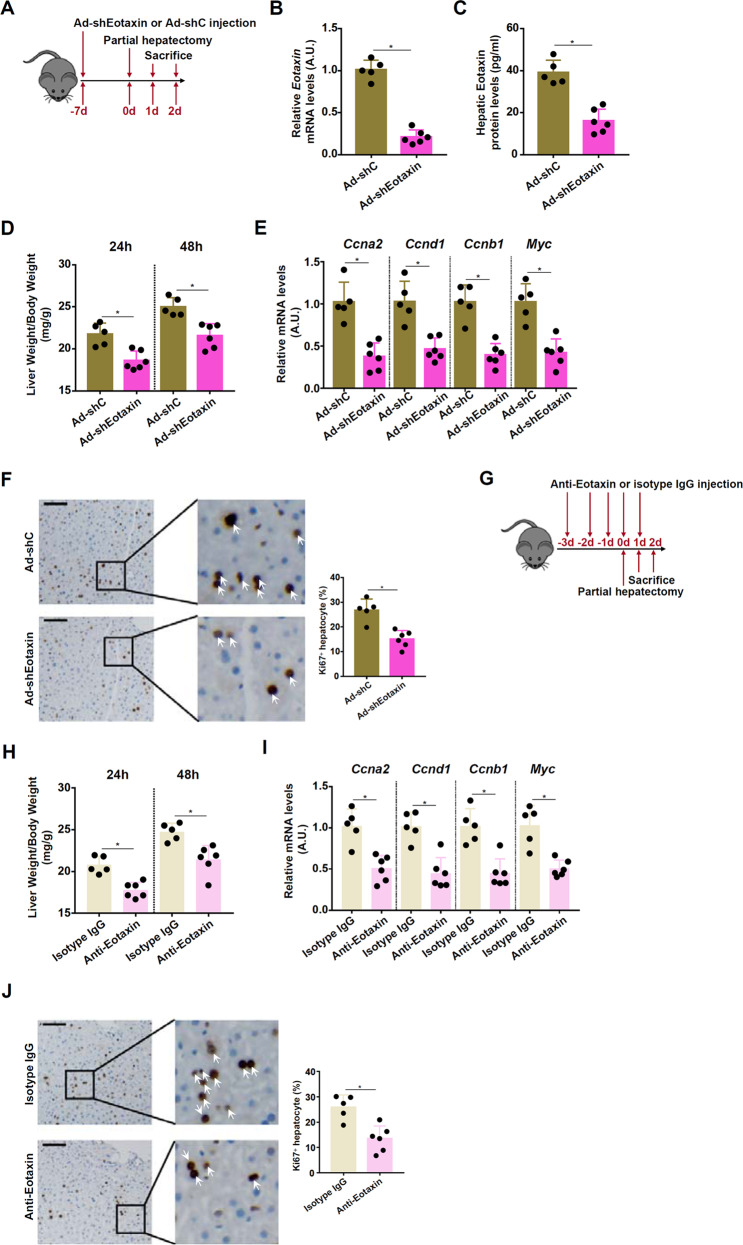
Eotaxin depletion impairs liver regeneration in mice. **A**–**F** C57BL/6 mice were injected with adenovirus carrying eotaxin shRNA vector (Ad-shEotaxin) or control adenovirus (Ad-shC) followed by 2/3 PHx (**A**). Hepatic eotaxin expression was examined by qPCR (**B**) and ELISA (**C**). Liver weight versus body weight was calculated at 24 and 48 h post-surgery (**D**). Expression levels of pro-proliferative genes were examined by qPCR (E) Paraffin embedded sections were stained with anti-Ki67 (F). *N* = 5–6 mice for each group. (**G-H**) C57BL/6 mice were injected with an eotaxin neutralizing antibody followed by 2/3 PHx (**G**). Liver weight versus body weight was calculated at 24 and 48 h post-surgery (**H**). Expression levels of pro-proliferative genes were examined by qPCR (**I**). Paraffin embedded sections were stained with anti-Ki67 (**J**). *N* = 5–6 mice for each group.

Two different strategies were exploited to examine the effect of eotaxin depletion on liver regeneration. In the first set of experiments, the mice were injected via tail vein adenovirus carrying an shRNA vector targeting eotaxin (Ad-shEotaxin) or a control shRNA vector (Ad-shC) prior to partial hepatectomy (Fig. 7A). RNAi-mediated knockdown lowered hepatic eotaxin mRNA (Fig. 7B) and eotaxin protein (Fig. 7C) levels. As a result, liver regeneration, as measured by post-surgery liver weight/body weight (Fig. 7D), expression levels of pro-proliferative genes (Fig. 7E), and Ki67 staining of proliferating hepatocytes (Fig. 7F), was significantly weakened. In the second set of experiments, the mice were injected with an eotaxin neutralizing antibody prior to partial hepatectomy (Fig. 7G). Eotaxin neutralization similarly retarded liver regeneration as evidenced by delayed recovery of liver mass (Fig. 7H), down-regulation of pro-proliferative genes (Fig. 7I), and reduced number of Ki67^+^ proliferating hepatocytes (Fig. 7J).

### Correlation between BRG1/EOTAXIN expression and eosinophil infiltration in human liver specimens

**Fig. 8:**
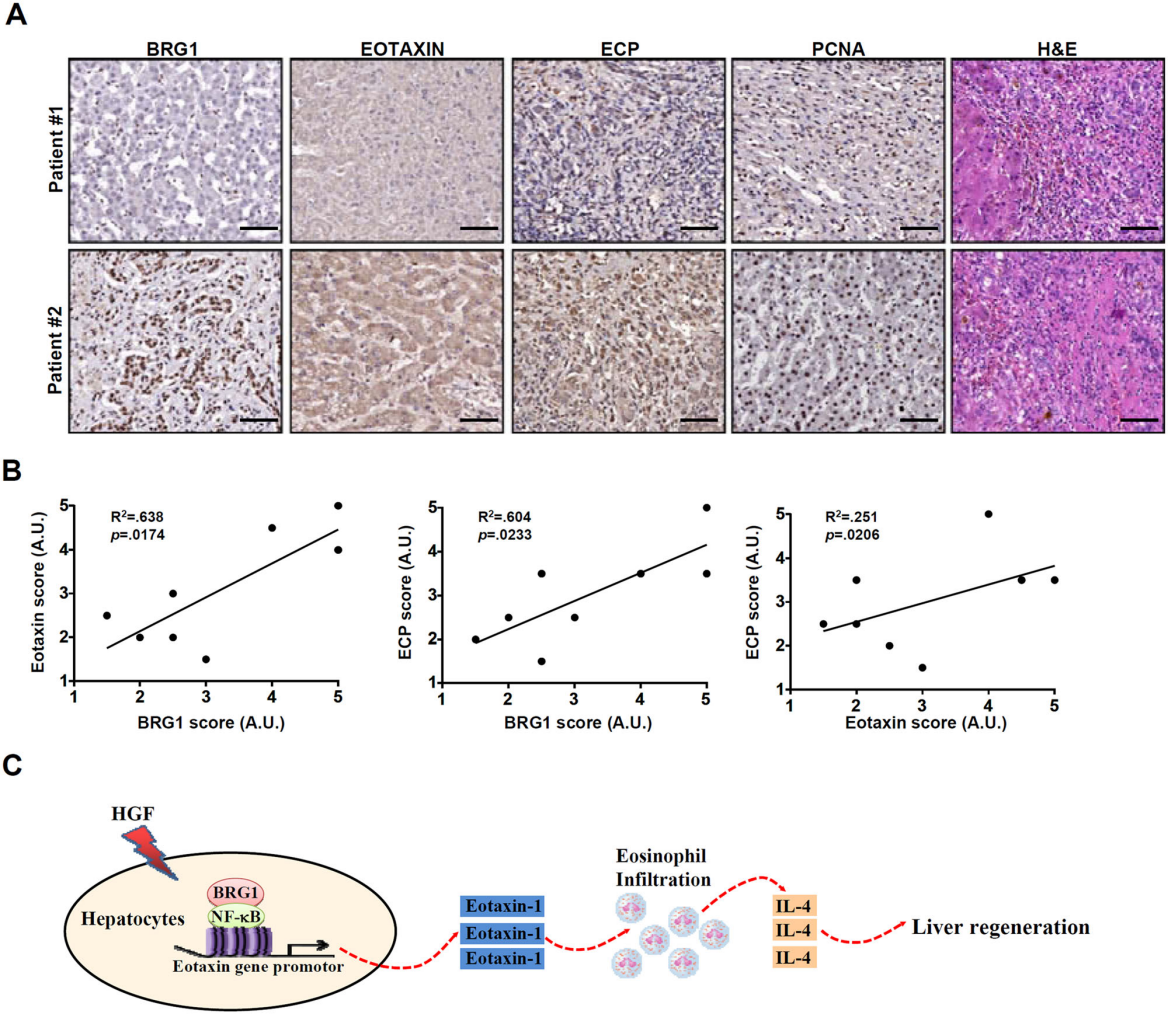
Correlation between BRG1/EOTAXIN expression and eosinophil infiltration in human liver specimens. **A** Paraffin sections of liver specimens collected from patients with acute liver failure were stained with anti-ECP, anti-Eotaxin, anti-BRG1, anti-PCNA, and H&E. *N* = 8 cases. **B** Linear regression was performed with Graphpad Prism. **C** A schematic model. In response to pro-regenerative stimuli (e.g., HGF) following liver injury, BRG1 interacts with NF-κB to activate eotaxin transcription in hepatocytes. Increased eotaxin levels promote the infiltration of eosinophils, which secrete pro-proliferative cytokines (e.g., IL-4) to stimulate liver regeneration.

In order to determine whether Brg1-mediated Eotaxin transcription might be involved in eosinophil infiltration in the human liver, we performed immunohistochemical staining using specimens from patients with acute liver failure. Levels of eotaxin and Brg1 were measured by histochemical staining with an anti-eotaxin antibody and an anti-Brg1 antibody, respectively. Eosinophil infiltration was measured by histochemical staining with an antibody that recognizes eosinophil cationic protein (ECP), a surface marker specific for eosinophils. The stainings were scored as described in the Methods: whereas the eotaxin score and the Brg1 score indicated their respective expression levels, the ECP score indicated the magnitude of eosinophil infiltration. As shown in Fig. 8A, strong eotaxin staining was evident where Brg1 expression was high and robust eosinophil infiltration was detected. Linear regression analyses confirmed that there was a significant correlation between BRG1/EOTAXIN expression and eosinophil infiltration in the human livers (Fig. 8B).

## Discussion

Liver regeneration is acutely influenced by the hepatic immune microenvironment [55–57]. The ΔdblGATA mice, which lacks the eosinophil lineage owing to the a mutation in the pioneering transcription factor GATA1, exhibit defective hepatocyte proliferation following partial hepatectomy alluding to a essential role for eosinophils in liver regeneration [17]. Eosinophils accumulate in the liver as hepatocytes re-enter cell cycle. Production and secretion of IL-4 is considered to a controlling step that mediates the regulatory effect of eosinophils. Indeed, mice with a deficiency in IL-4 fail to regenerate the liver following hepatectomy [58]. On the contrary, supplementation of exogenous IL-4 is sufficient to normalize the regenerative response of liver in the ΔdblGATA mice [17]. Here we demonstrate that elevation of eotaxin, an eosinophil-specific chemokine, represents an early event in the course of liver regeneration. The functional relevance of this finding, however, remains to be determined. It is noteworthy that other cell lineages, in addition to eosinophils, can be navigated by eotaxin. Salcedo et al have shown that eotaxin promotes angiogenesis by engaging its receptor CCR3 expressed on endothelial cell surface [59]. Eotaxin can also steer the movement of basoniphil [60], fibroblast [61], and neutrophils [62]. The increased presence of these cell types has been observed in the regenerating liver and is functionally pertinent. We show here that eotaxin depletion compromised liver regeneration in mice. However, it is not immediately clearly whether this was due to an alteration of eosinophil infiltration and/or intrahepatic IL-4 signaling.

We show here Brg1 is responsible for eotaxin induction in hepatocytes in response to pro-regenerative cues. Further, supplementation of exogenous eotaxin, at least in part, normalized the impairment of liver regeneration in Brg1^LKO^ mice. This observation adds an additional layer of regulation by which Brg1 contributes to recruitment of immune lineages to impact tissue injuries. We have previously shown that Brg1 activates the transcription of SPON2 [46] and MRP8 [40], respectively, in vascular endothelial cells to promote macrophage trafficking. More importantly, Brg1 can induce the production of multiple hepatocyte-derived chemoattractants. Li et al have demonstrated that galectin-3 (LGALS3) expression is severely disturbed in Brg1-null hepatocytes exposed to injurious cues [63]. Galectin-3 has a well-documented role in chemotaxis being able to impact the migration of several different immune lineages including macrophages [64], neutrophils [65], dendritic cells [66]. Galectin-3 deficiency impairs liver regeneration in mice in a diet-induced model of liver injury although it is not clear whether there is a concomitant alteration of immune cell infiltration in the livers of these animals [67]. Of interest, Rao et al have observed that galectin-3 promotes the rolling and adhesion of eosinophils in a cell-autonomous manner thus aiding their mobilization [68] pointing to the possibility that Brg1 loss-of-function might contribute to altered eosinophil migration as a result of galectin-3 deficiency. Alternatively, Hong et al have recently reported that Brg1-dependent transcription of nephronectin (Npnt) mediates T lymphocyte recruitment in a mouse model of fulminant hepatitis [33]. A growing body of evidence supports a pivotal for T lymphocytes in the regulation of liver regeneration [69–72]. Thus, Brg1 could potentially modulate intrahepatic T cell population contributing to liver regeneration. Of note, these putative scenarios with regard to Brg1-mediated immune cell trafficking in the course of liver regeneration are not necessarily mutually exclusive because different immune cell lineages form extensive crosstalk. The new observation presented here broadens our understanding on the connection between Brg1 and hepatic immunity but clearly demands further investigations into the underlying mechanisms.

Our data show that Brg1 is recruited by NF-κB to the eotaxin promoter. Of interest, we have previously shown that Brg1 can be recruited by NF-κB to the pro-inflammatory mediator promoters including TNF-α and IL-6in the context of non-alcoholic steatohepatitis [27]. Both TNF-α [73] and IL-6 [74] play essential roles in liver regeneration raising the possibility that Brg1 may rely on NF-κB to regulate the expression of multiple pro-regenerative cytokines to promote liver regeneration. NF-κB is subjected to a range of different post-translational modifications including phosphorylation, methylation, and acetylation [75]. Whether there exists a specific barcode of NF-κB modifications that determines the locus-specific recruitment of Brg1 to these pro-regenerative target promoters is yet another open question. In addition, Brg1 typically functions in the context of a mega-protein complex. Previously, Sinha et al have reported that Brg1 up-regulation precedes that of Brm in a model of liver injury-regeneartion [76]. Because both Brg1 and Brm can interact with NF-κB [77], the lingering question as to whether Brm can be recruited by NF-κB to specific promoters and participate in liver regeneration needs to be thoroughly investigated in the future.

Our data also indicate that NF-κB knockdown or inhibition attenuates eotaxin transcription in hepatocytes likely by blocking Brg1 recruitment, which points to a role for NF-κB in liver regeneration. Germline deletion of NF-κB/p65 in mice causes embryonic lethality due to TNF-α induced apoptosis. Spatiotemporal manipulations of NF-κB/p65 expression and/activity have led to, however, conflicting results. Over-expression of a NF-κB/p65 super-repressor (IκB-SR) by adenovirus in adult animals disrupts cell cycle progression of hepatocytes and exacerbates liver regeneration following partial hepatectomy, suggesting that NF-κB/p65 is essential for the liver regenerative response [78]. Consistently, mice harboring knock-in alleles of NF-κB/p65^T505A^ in which the threonine 505 was substituted with an alanine thus blocking the repressive phosphorylation of this residue display stronger proliferation of hepatocytes compared to the controls following partial hepatectomy [79]. On the contrary, conditional deletion of NF-κB/p65 in hepatocytes does not appear to delay or enhance liver regeneration [80]. In addition, deletion of p50, the NF-κB subunit typically found in a heterodimer with p65, proves to be dispensable for liver regeneration [81]. Moreover, both IκB kinase α (IKKα), the canonical activator of the NF-κB pathway and NF-κB-inducing kinase (NIK), the non-canonical activator of the NF-κB pathway, suppress liver regeneration in mice [82]. None of these reports examined the effects of NF-κB manipulation on eosinophils in the liver. Adding to the complexity of this issue is the observation that NF-κB/p65 represses IL-4 transcription by competing with NFAT to bind to the IL-4 promoter [83]. Whether transcriptional repression of IL-4 by NF-κB/p65 could be offset by transcriptional activation of eotaxin and subsequent eosinophil attraction remains to be determined in future studies. Curiously, although both eotaxin-2/CCL24 and eotaxin-3/CCL26 have been shown to promote eosinophil chemotaxis, neither appears to be altered during liver regeneration (Fig. 1). The CCL26 promoter contains a conserved NF-κB motif that mediates its up-regulation by combined treatment with IL-4 and TGF-β in airway smooth muscle cells [41] where the molecular structure of the CCL24 promoter remains to be characterized. The fact that eotaxin, but not eotaxin-2 or eotaxin-3, is up-regulated during liver regeneration suggests that NF-κB is not the rate-limiting factor. The mechanism whereby one of the three eotaxin members is selectively activated awaits further investigation.

In conclusion, we provide evidence to show that eosinophil-specific chemokine eotaxin is up-regulated at the transcriptional level in the course of liver regeneration in vivo and in vitro. Future studies should strive to tackle the following critical questions: Whether supplementation of IL-4, the supposedly mandatory mediator of eosinophil-associated functions, could rescue eotaxin deficiency? Supplementation of eosinophils and eosinophil-derived substances has been demonstrated to confer protection in a number of disease models. Addressing these lingering issues in future studies would be important for the consideration of exploiting similar strategies in the treatment of liver failure.

## Supplementary information

checklist

online supplementary material

## Data Availability

The data that support the findings of this study are available upon reasonable request.
