# Supplementary material for: Trans-activation of eotaxin-1 by Brg1 contributes to liver regeneration
Source: Cell Death Dis. 2022 May 25;13(5):495. doi: 10.1038/s41419-022-04944-0 (PMC9132924; doi:10.1038/s41419-022-04944-0)
Supplement: Supplementary file 2 — online supplementary material [file 41419_2022_4944_MOESM2_ESM.docx]

**Fan ZW et al: Trans-activation of eotaxin-1 by Brg1 contributes to liver regeneration**

**Online supplementary material**

**Fig.S1: Uncropped blots.**

**Table I: ALT Patient Information Sheet**

| **Patient ID** | **Gender** | **Age**  **(yr)** | **BT**  **(^o^C)** | **BP**  **(mmHg)** | **ALT**  **(U/dL)** | **AST**  **(U/dL)** | **LDH**  **(U/dL)** | **Bilirubin**  **(μM)** |
| --- | --- | --- | --- | --- | --- | --- | --- | --- |
| 1 | Female | 30 | 36.8 | 102/71 | 547.3 | 547.7 | 890 | 364.2 |
| 2 | Male | 29 | 36.9 | 118/76 | 1687 | 633 | 272 | 529.1 |
| 3 | Male | 26 | 36.3 | 110/70 | 582.8 | 1790.6 | 1684 | 364.5 |
| 4 | Female | 25 | 36.5 | 100/70 | 2344 | 835 | 284 | 95.9 |
| 5 | Female | 44 | 37.0 | 122/78 | 1525 | 1460 | 881 | 461.7 |
| 6 | Male | 42 | 36.8 | 103/58 | 1726 | 1123.9 | 2815 | 157.3 |
| 7 | Male | 29 | 36.4 | 123/84 | 729 | 174.8 | 346 | 298.7 |
| 8 | Male | 44 | 36.0 | 132/79 | 587 | 728 | 913 | 461.7 |

| **Patient**  **ID** | **Gender** | **Age**  **(y)** | **Temp**  **(^o^C)** | **BP**  **(mmHg)** | **ALT**  **(U/dL)** | **AST**  **(U/dL)** | **LDH**  **(U/dL)** |
| --- | --- | --- | --- | --- | --- | --- | --- |
| 1 | M | 42 | 36.8 | 103/58 | 1726 | 1124 | 2815 |
| 2 | M | 29 | 36.4 | 123/84 | 729 | 175 | 346 |
| 3 | F | 30 | 36.8 | 102/71 | 547 | 548 | 890 |
| 4 | M | 29 | 36.9 | 118/76 | 1687 | 633 | 272 |
| 5 | M | 26 | 36.3 | 110/70 | 583 | 1791 | 1684 |
| 6 | M | 63 | 36.2 | 145/75 | 1598 | 1578 | 8870 |
| 7 | M | 44 | 36.0 | 132/79 | 587 | 728 | 913 |
| 8 | F | 44 | 37.0 | 122/78 | 1525 | 1460 | 881 |
